# c.151dup variant in *LAMA*3 in Pakistani patients affected with Shabbir Syndrome but showing mild symptoms

**DOI:** 10.12669/pjms.39.4.6926

**Published:** 2023

**Authors:** Asmat Ullah, Fibhaa Syed, Shazia Khan

**Affiliations:** 1 Asmat Ullah Department of Biochemistry Faculty of Biological Sciences, Quaid-i-Azam University, Islamabad, Pakistan; 2 Fibhaa Syed Department of General Medicine, Shaheed Zulfiqar Ali Bhutto Medical University, PIMS, Islamabad, Pakistan; 3 Shazia Khan Department of Biological Sciences, International Islamic, University Islamabad, Pakistan, Hafeez Institute of Medical Sciences Islamabad, Pakistan

**Keywords:** Shabbir Syndrome, LOC Syndrome, c.151dup, LAMA3, Diagnostic Marker

## Abstract

**Objective:**

To identify genetic causes of Shabbir syndrome in two patients of Pakistani origin.

**Methods:**

In the present study, we have investigated a Pakistani family with two affected members segregating Laryngo-onycho-cutaneous (LOC) syndrome. The patients were diagnosed as suspected cases of LOC based on phenotypes including abnormal larynx, nails, and hyperpigmentation in patients’ eyes. Genetic investigation was done by performing whole exome sequencing (WES) using DNA of the patients. Sanger sequencing was performed to validate WES findings and segregation analysis in the family.

**Results:**

Data analysis of exomes and Sanger sequencing of patients revealed a homozygous one base pair duplication (NM_000227.6; *LAMA3*; c.151dup; p.Val51GlyfsTer4) in *LAMA3* in the patients. Parents of the patients were heterozygous for the identified variant.

**Conclusion:**

Previously, the same variant has been found in most of the Pakistani Punjabi patients affected with LOC. Therefore, Pakistani Punjabi families affected with Shabbir Syndrome may be screened for c.151dup variant in *LAMA3* using targeted sequencing. Sanger sequencing is a cost-effective and time-saving technique as compared to whole exome/genome sequencing. Hence, developing ethnicity-specific *LAMA3* targeted molecular diagnostic test would be cost-effective. Further, the study would assist in carrier testing and prenatal diagnosis of the affected families.

## INTRODUCTION

Among inherited skin disorders, Laryngo-onycho-cutaneous (LOC) (OMIM 245660) syndrome also known as Shabbir syndrome is categorized under junctional epidermolysis bullosa (JEB).^1^,^2^ Here, laryngo refers to the disorder involving larynx and respiration, onycho means the abnormal development of nails and cutaneous meaning the defects in skin or its appendages. So, collectively larynx, nails and skin are mainly affected in this syndrome. LOC was first identified by a Pakistani clinician Shabbir in 1986 in the children born to consanguineous parents, originating from the Pakistani Punjabi Muslim population.^1^

The early onset of this syndrome is characterized by a hoarse cry soon after birth, development of granulated tissue in nails bed, mucosal regions, larynx and ocular region, delayed tooth enamel formation.^1^-^6^ Later, granulation tissue may also develop in epiglottis and trachea, and in severe cases death occur because of difficulty in breathing.^7^ McLean et al.^7^ identified the molecular basis for LOC syndrome and linked this disease to Laminin Alpha-3 (*LAMA-3*) by homozygosity mapping.^7^ *LAMA3* instruct the formation of one of the subunits (alpha subunit) of Laminin 332 protein, which is made up of three subunits i.e., alpha, beta and gamma.^8^

This laminin protein help attach the epidermis of the skin to the underlying layers, thus playing an important role in stabilizing and strengthening the skin. In addition, it is proposed that laminin 332 protein have roles in the development of cornea (outer layer of eyes) and tooth enamel.^9^,^10^ Any pathogenic variant in *LAMA3* gene leads to either absence or formation of mutated LAMA3 protein, thus leading to poor attachment of skin layers, causing skin erosions and development of granulation tissues, characteristic of LOC syndrome.^11^-^15^ In the present study, two patients segregating LOC-like phenotypes in autosomal recessive form were clinically diagnosed. Whole exome sequencing followed by Sanger sequencing and pathogenicity calculations were performed to understand pathomechanism of the disease phenotypes.

## METHODS

The present study demonstrated a consanguineous Pakistani family of Punjabi origin, displaying the characteristics of LOC syndrome. After informed written consents of the participating individuals, the study was approved from the Institutional Review Board (protocol # IRB-QAU-131) of the Quaid-e-Azam University, Islamabad, Pakistan. Clinically, the patients were diagnosed at the department of general medicine in Pakistan Institute of Medical Sciences (PIMS) Islamabad, Pakistan. The patients were recruited from PIMS during December 2018-2021.

### DNA Extraction and Whole Exome Sequencing:

DNA was extracted from the blood samples of affected and unaffected individuals of the family by using Phenol–chloroform extraction protocol. DNA was quantified using spectrophotometry. Whole exome sequencing was performed using DNA from two affected individuals (IV-3 and IV-4) on an Illumina HiSeq 2500 sequencer. Libraries were prepared using Agilent SureSelect Target Enrichment Kit by following the manufacturer’s instructions. Reads were analyzed using the BWA Enrichment application of BaseSpace (Illumina Inc. 5200 Illumina Way | San Diego, CA, 92122, USA). Alignment of the reads with reference genome was performed with Burrows-Wheeler Aligner (BWA). Variants were called with the Genome Analysis Toolkit (GATK). All the called variants were annotated with wANNOVAR (https://wannovar.wglab.org/index.php).

### WES Data Analysis:

WES data was searched for pathogenic variants shared by both the affected individuals. Variants having minor allele frequency higher than one percent in any of human genome databases (1000 Genomes Project, Genome Aggregation Database (gnomAD), Exome Aggregation Consortium (ExAC), Exome Sequencing Project 6500 (ESP6500), and database of single nucleotide polymorphisms (dbSNP) were excluded from downstream analysis. Only the non-synonymous and splice site variants were prioritized for further analysis. Based on autosomal recessive inheritance of the disease due to consanguineous union between parents of the patients, we prioritized homozygous variants in the exomes. Variants that were predicted benign or tolerated by human mutation prediction tools (MutationTaster, SIFT, PolyPhen-2, CADD, varSEAK and varsome among others) were excluded from the list of prioritized variants.

### Sanger Sequencing:

To validate WES data and check the segregation of prioritized variant in LAMA3 in the family, Sanger sequencing was performed using DNA of parents and normal siblings of the patients. Reference sequence of *LAMA3* was downloaded from Ensemble genome browser (https://www.ensembl.org/index.html). Primers sequences used for the amplification of the identified variant were Forward = 5’-CCTGTGATTTAGGGCGTCTG-3’ and Reverse = 5’-CCTCAGTCCACCCATTTACTC-3’. The designed primers were amplified using Polymerase Chain Reaction (PCR). PCR was carried out in 25 μL reaction volumes containing 40 ng genomic DNA, 20 pmol of each primer, 200 mM of each deoxynucleoside triphosphate, 2.5 μL reaction buffer (MBI Fermentas, Life Sciences, York, UK), 2.5 μL MgCl_2_ (1.5 mM) and one unit Taq DNA polymerase (MBI Fermentas). The PCR conditions used were 96°C for one min, followed by 39 cycles of 96°C for 45 sec, 59°C for 45 sec, 72°C for 45 sec, and a final extension at 72°C for ten minutes.

PCR-amplified products were purified with commercially available kits (Axygen, CA, USA) and sequencing was performed with the BigDye Terminator v3.1 Cycle Sequencing Kit, on ABI Prism 310 Genetic Analyzer (Applera, Foster City, CA, USA). Bioedit was used to align the sequences in order to find out the variant. American College of Medical Genetics (ACMG) classification (http://wintervar.wglab.org/results.php) was followed to interpret the identified variant.

## RESULTS

**Fig.1:**
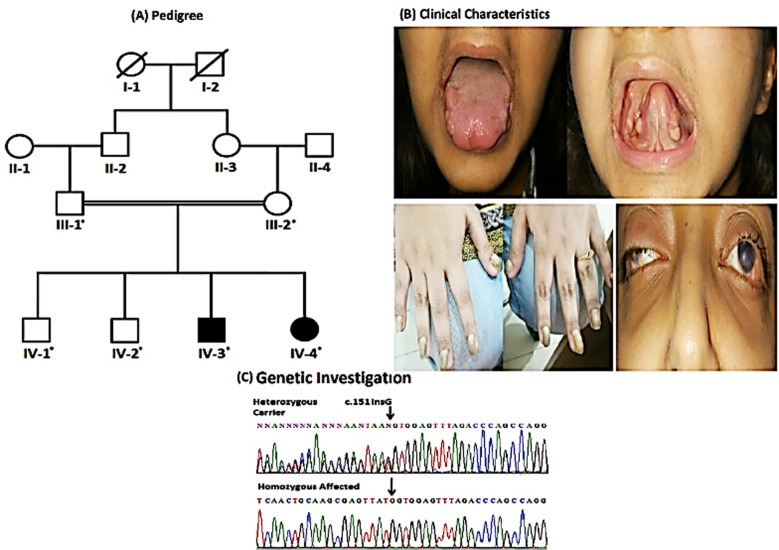
four-generation pedigree of the present family showing autosomal recessive inheritance pattern of the disease. Males and females are represented with squares and circles respectively. Filled symbols represent affected while unfilled symbols are the representative of healthy individuals. Asterisks above symbol represent availability of the individual for present study. Double line between male and female shows consanguinity among parents (Fig1A). Clinical phenotypes of the patient (IV-4) showing development of granulated tissue in nail bed, oral mucosal cavity and ocular region (Fig.1B). Partial sequence of *LAMA3* showing c.151dup variant in patients (homozygous) and their parents/siblings (heterozygous). Insertion/duplication at nucleotide position 151 is shown with an arrow (Fig.1C).

In the present study we recruited two patients (IV-3 and IV-4) afflicted with LOC (Fig.1A). Patient IV-3 was 31 years old male at the time of participation in the present study. He has another affected sibling IV-4 who was 16 years old at the time of study (Fig.1B).

Clinical features of the patients were in accordance with that of LOC syndrome. Both patients were having granulation tissues in the eyes, precisely the conjunctiva and also on the tongue and larynx. Teeth were normal but nails were also deformed with the development of granulated tissues under the nail beds. Nails were thickened and slow in regrowth (Fig.1B). Patients didn’t respond to any drugs including antibiotics and dapsone. Both the affected individuals were having normal intellectual ability and were able to perform their daily life activities. The patients did not show airway obstruction.

### Molecular analysis:

Whole exome sequencing of each individual revealed approximately 95 to 98 thousand variants. In total, 179 homozygous variants shared by both the affected individuals were found. Data analysis of whole exome sequencing revealed a homozygous one base pair duplication (c.151dup; p.Val51GlyfsTer4) in *LAMA3* shared by both affected individuals (Fig.1C). Sanger sequencing validated the WES findings and correct segregation of the variant with the disease phenotypes in family members. Both the patients were homozygous while parents and normal siblings of the patients were heterozygous for c.151dup. The pathogenicity was supported by MutationTaster, SIFT, CADD, varsome and ACMG (PV1, PP5, PM2). The variant was not found in homozygous state in 50 exomes of healthy controls of same ethnicity.

## DISCUSSION

The present study reports clinical and genetic characterization of a Pakistani family unrelated to the previously reported families segregating LOC syndrome. Clinical investigation of the present patients revealed similar phenotypes of LOC previously reported in the families of Punjabi population, but with variable severity. WES and Sanger sequencing identified a homozygous pathogenic one base pair duplication (c.151dup) in *LAMA3* in the present affected individuals. Previously, genetic analysis of LOC families originating from Pakistan revealed a homozygous duplication variant (c.151dup; p.Val51GlyfsTer4) in *LAMA3* in six unrelated families.^7^,^14^ Patients studied by McLea et al.^7^ showed failure of tooth enamel formation and marked dental malformations but the patients in present study showed normal teeth. Similarly, patients reported by Khan et al.^14^ showed airway obstruction that was not observed in the present patients.

No respiratory complications were observed in our patients as compared to patients reported by McLea et al.^7^ that were reported to die in childhood due to acute or chronic respiratory obstruction with secondary pulmonary sepsis. Elder patients in the cohort studied by McLea et al.^7^ were blind due to corneal pterygium formation while the present patients were able to see, read and perform daily routine activities. Nails dystrophy was more severe in present patients as compared to those studied by McLea et al.^7^ The intra-familial variability in phenotypes might be due to different familial background of the affected families, where SNPs in the modifier genes have their effect on differential phenotypes.

The LOC was first clinically characterized by Shabir in 1986 in 22 patients from 12 families of Pakistani origin. Genetic causes of the syndrome were reported by McLean et al.^7^ in six families of Punjabi ethnicity in Pakistan. At the time, all LOC families reported were of Punjabi origin, suggesting a founder effect as this disorder was not seen in other populations.^7^ LOC syndrome is a rare autosomal recessive disorder that primarily affects families of Punjabi background from India and Pakistan. Although the development of granulation tissue was not mentioned in the first report but it remained consistent in all the patients in subsequent reports.^3^-^5^ In severe cases, death occurs but those who survive have marked dental deformities, and symptoms also slowly improve with age.

The variant c.151dup in *LAMA3* has been exclusively seen in Pakistani population showing its founder effect. The identified variant in *LAMA3* causes a frameshift in the reading frame and leads to the formation of a truncated protein as amino acid valine at position 51 is changed to glycine. It culminates in generation of termination code after four nucleotides leading to an abnormal protein product (p.Val51GlyfsTer4). McLean et al.^7^ found that duplication of guanine at nucleotide position 151 of *LAMA3* does not result in nonsense-mediated mRNA decay due to rescue of the transcript by an alternative translation start site six exons downstream. This will result in N-terminal deletion of laminin alpha 3a generating a deficiency of laminin 332, ultimately leading to the poor skin layers attachment and development of granulation tissue characterizing LOC syndrome.^7^

Till now, only five variants in *LAMA3* underlying LOC syndrome have been identified in different ethnicities (http://www.hgmd.cf.ac.uk/ac/index.php),^16^-^18^ of which c.151dup has been reported to segregate in most of the cases. Mclean et al.^7^ reported six families of Punjabi ethnicity of Pakistan origin segregating common variant (c.151dup) underlying LOC syndrome. Figueira et al.^19^ reported an Irish LOC patient segregating p.I17N variant in LAMA3. Barzegar et al.^20^ reported a family of Iranian origin afflicted with LOC due to homozygous variants (p.Gln57X) in the *LAMA3*. Recently, Ranugha et al.^21^ reported an Indian Punjabi family affected with LOC due to a homozygous frameshift variant (p. Ser1967GlnfsTer5) in *LAMA3*. More recently, Prodinger et al.^17^ studied six new cases of LOC of Pakistani (n = 2), Estonian (n = 1), Black African (n = 1), and Middle Eastern (n = 2) origin and reported a novel c.6612_6615delCCAG and a reported c.151dup variant in *LAMA3* as underlying causes of the disease.

### Limitation:

As our study only reports single pathogenic variant in *LAMA3*, therefore, it could be possible that some of the patients did not carry the specific variant. In that case, whole *LAMA3*, whole exome or genome sequencing will be performed, which could be a limitation of the present study. Only one family was studied in the present study, including a large cohort of similar phenotypes would be helpful in making correct decision for declaring c.151dup as a diagnostic marker.

## CONCLUSION

In total, 27 LOC cases have been reported till date. Among these LOC cases, most of them are from the Punjabi Muslim population in Pakistan and India due to c.151dup variant in *LAMA3* (Supplementary Table-I). Thus, it can be concluded that the variant has founder effect in this specific population. Therefore, we recommend screening LOC patients of Pakistani ethnicity for c.151dup through Sanger sequencing using variant specific primers. Sanger sequencing is a cost-effective and time-saving technique as compared to whole exome/genome sequencing. Hence, developing ethnicity-specific *LAMA3* targeted molecular diagnostic test would be cost-effective.

**Supplementary table 1:** List of pathogenic mutations in LAMA3 underlying LOC.

Mutation	Phenotype	Ethnicity	Reference
c.151dup	Both patients were having granulation tissues in the eyes, precisely the conjunctiva and on the tongue and larynx with normal teeth, deformed nails with the development of granulated tissues under the nail beds. Nails were thickened and slow in regrowth.	Punjabi, Pakistan	Present study
c.47G>A, p.W16*)	Severe fingernail onychodystrophy; extensive mutilating erosions and scars involving extremities and hypertrophic scars in elbows, and orofacial regions the armpit , severe enamel hypoplasia, pitted teeth, hypodontia.	Chinese	Wang et al., 2022
c.151insG	Facial erosion and subungual granulation tissue along with severe hoarseness of voice at the age of six months. The formation of granulation tissue under the toenails, thickened nails, bilateral conjunctival masses and tooth anomalies airway obstruction.	Punjabi, Pakistan	Khan et al., 2021
p.Gln57X	Prominent granulation tissue affecting the right eye margin leading to loss of vision, small teeth with hypoplastic enamel. Fingernail dystrophy with periungual inflammation is present. Discrete hypertrophic scars especially on lower limbs legs and knees.	Iran	Barzegar et al., 2013
IVS34+1G>A and p.Iso17Asn	periungual blisters, oral mucosal erosions, and hoarseness due to laryngeal blisters and ulcerations with facial erosions and granulation tissue in the margin of the left upper marginal eyelid. Granulation tissue in the left eyelid. Exuberant granulation tissue in the substance of the nodules.	Caucasian	Figueira et al., 2007
c.151insG	Hoarse cry in first few weeks of life or evidence of laryngeal granulation tissue, scarring or stenosis in the first few years of life; nail dystrophy; ocular involvement including aggressive conjunctival pannus formation; dental anomalies (hypodontia, poor enamel formation, excessive caries); slow-healing cutaneous erosions; absence of a history of skin fragility or blistering at any stage.	Muslim population in Punjab region of Pakistan and India	McLean et al., 2003

The consanguineous marriages are very common in Pakistani population, as there is more than 62% consanguinity in the population.^22^-^23^ Due to higher rate of consanguinity in the population, the heterozygous variant c.151dup in *LAMA3* has higher probability to become homozygous in upcoming generations. To prevent the segregation of the mutated allele in homozygous state and disease phenotypes to the next generations, normal individuals of LOC-affected families can be tested for carrier screening. Thus, the study will also help in the carrier screening, genetic counseling and prenatal testing of the families having positive history of LOC syndrome.

### Author’s Contributions:

**AU:** Recruited samples and collected/compiled clinical information with help of **SK** and **FS**. **FS:** Clinically characterized the patients. **AU, SK:** Performed genetic studies, analyzed the data, and wrote manuscript. All the authors critically revised the manuscript and contributed to the discussion and are responsible and accountable for the accuracy or integrity of the work.

## References

[1] Shabbir G, Hassan M, Kazmi A (1986). Laryngo-onycho-cutaneous syndrome:a study of 22 cases. Biomedica.

[2] Fine JD, Eady RA, Bauer EA, Bauer JW, Bruckner-Tuderman L, Heagerty A (2008). The classiﬁcation of inherited epidermolysis bullosa (EB):report of the Third International Consensus Meeting on Diagnosis and Classiﬁcation of EB. J Am Acad Dermatol.

[3] Phillips RJ, Atherton DJ, Gibbs ML, Strobel S, and Lake BD (1994). Laryngo-onycho-cutaneous syndrome:an inherited epithelial defect. Arch Dis Child.

[4] Ainsworth JR, Spencer AF, Dudgeon J, Geddes NK, Lee WR (1991). Laryngeal and ocular granulation tissue formation in two Punjabi children:LOGIC syndrome. Eye.

[5] Ainsworth JR, Shabbir G, Spencer AF, Cockburn F (1992). Multisystem disorder of Punjabi children exhibiting spontaneous dermal and submucosal granulation tissue formation:LOGIC syndrome. Clin Dysmorph.

[6] Romanos GE, Slots J, Javed F (2013). Aggressive periodontitis in a young Pakistani female with laryngo-onycho-cutaneous syndrome. J Oral Sci.

[7] McLean WH, Irvine AD, Hamill KJ, Whittock NV, Coleman-Campbell CM, Mellerio JE (2003). An unusual N-terminal deletion of the laminin alpha3a isoform leads to the chronic granulation tissue disorder laryngo-onycho-cutaneous syndrome. Hum Mol Genet.

[8] Vidal F, Baudoin C, Miquel C, Galliano MF, Christiano AM, Uitto J (1995). Cloning of the laminin alpha-3 chain gene (LAMA3) and identification of a homozygous deletion in a patient with Herlitz junctional epidermolysis bullosa. Genomics.

[9] Barrera V, Troughton LD, Iorio V, Liu S, Oyewole O, Sheridan CM (2018). Differential Distribution of Laminin N-Terminus α31 Across the Ocular Surface:Implications for Corneal Wound Repair. Invest Ophthalmol Vis Sci.

[10] Dinckan N, Du R, Petty LE, Coban-Akdemir Z, Jhangiani SN, Paine I (2018). Whole-Exome Sequencing Identifies Novel Variants for Tooth Agenesis. J Dent Res.

[11] Yenamandra VK, Vellarikkal SK, Kumar M, Chowdhury MR, Jayarajan R, Verma A (2017). Application of whole exome sequencing in elucidating the phenotype and genotype spectrum of junctional epidermolysis bullosa:A preliminary experience of a tertiary care centre in India. J Dermatol Sci.

[12] Ahmad F, Shah K, Umair M, Jan A, Irfanullah, Khan S (2018). Novel autosomal recessive LAMA3 and PLEC variants underlie junctional epidermolysis bullosa generalized intermediate and epidermolysis bullosa simplex with muscular dystrophy in two consanguineous families. Clin Exp Dermatol.

[13] Herrmann I, Linder KE, Meurs KM, Friedenberg SG, Cullen J, Olby N (2021). Canine junctional epidermolysis bullosa due to a novel mutation in LAMA3 with severe upper respiratory involvement. Vet Dermatol.

[14] Khan FF, Khan N, Rehman S, Ejaz A, Ali U, Erfan M (2021). Identification and Computational Analysis of Novel Pathogenic Variants in Pakistani Families with Diverse Epidermolysis Bullosa Phenotypes. Biomolecules.

[15] Li C, Liu L, Ma J, Zu J, Ma X, Sun M (2022). A pedigree study of laryngo-onycho-cutaneous syndrome with a novel mutation on LAMA3 gene. Zhonghua Er Bi Yan Hou Tou Jing Wai Ke Za Zhi.

[16] Vodo D, Malchin N, DeRowe A, Sprecher E, Sarig O (2021). Atypical presentation of laryngo-onycho-cutaneous syndrome resulting from novel mutations in LAMA3A. Clin Exp Dermatol.

[17] Prodinger C, Chottianchaiwat S, Mellerio JE, McGrath JA, Ozoemena L, Liu L (2021). The natural history of laryngo-onycho-cutaneous syndrome:A case series of six pediatric patients and literature review. Pediatr Dermatol.

[18] Wang R, Sun L, Habulieti X, Liu J, Guo K, Yang X, Ma D, Zhang X (2022). Novel variants in LAMA3 and COL7A1 and recurrent variant in KRT5 underlying epidermolysis bullosa in five Chinese families. Front Med.

[19] Figueira EC, Crotty A, Challinor CJ, Coroneo MT, Murrell DF (2007). Granulation tissue in the eyelid margin and conjunctiva in junctional epidermolysis bullosa with features of laryngo-onycho-cutaneous syndrome. Clin Exp Ophthalmol.

[20] Barzegar M, Mozafari N, Kariminejad A, Asadikani Z, Ozoemena L, McGrath JA (2013). A new homozygous nonsense mutation in LAMA3A underlying laryngo-onycho-cutaneous syndrome. Br J Dermatol.

[21] Ranugha P, Shastry V (2020). A novel mutation in LAMA3A gene in a child with laryngo-onycho-cutaneous syndrome from the Indian subcontinent. Indian J Dermatol Venereol Leprol.

[22] Umair M, Ahmad F, Ullah A (2018). Whole exome sequencing as a diagnostic tool for genetic disorders in Pakistan. Pak J Med Res.

[23] Majeed AI, Ullah A, Jadoon M, Ahmad W, Riazuddin S (2020). Screening, diagnosis and genetic study of breast cancer patients in Pakistan. Pak J Med Sci.

